# Protocol for training MERGE: A federated multi-input neural network for COVID-19 prognosis

**DOI:** 10.1016/j.xpro.2023.102812

**Published:** 2024-01-04

**Authors:** Bruno Casella, Walter Riviera, Marco Aldinucci, Gloria Menegaz

**Affiliations:** 1 Computer Science Department, University of Turin, 10149 Turin, Italy; 2 Computer Science Department, University of Verona, 37134 Verona, Italy; 3 Engineering for Innovation Medicine Department, University of Verona, 37134 Verona, Italy

**Keywords:** Bioinformatics, Health Sciences, Clinical Protocol, Computer sciences

## Abstract

Federated learning is a cooperative learning approach that has emerged as an effective way to address privacy concerns. Here, we present a protocol for training MERGE: a federated multi-input neural network (NN) for COVID-19 prognosis. We describe steps for collecting and preprocessing datasets. We then detail the process of training a multi-input NN. This protocol can be adapted for use with datasets containing both image- and table-based input sources.

For complete details on the use and execution of this protocol, please refer to Casella et al.^1^

## Highlights

• Train a multi-input neural network exploiting different types of input data
• Steps to train in both a typical machine learning setting and a federated scenario
• The combined use of different types of data is beneficial to the federated model
• This protocol shows how to train a federated multi-input neural network with OpenFL

## Abstract (editor highlights)

Publisher’s note: Undertaking any experimental protocol requires adherence to local institutional guidelines for laboratory safety and ethics.

## Graphical abstract

**Figure:**
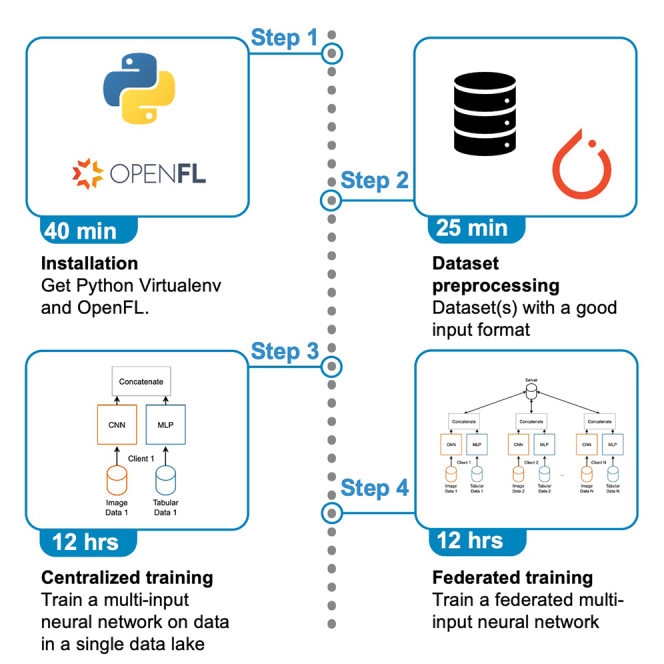

## Before you begin

**Timing: 40 min**

This section includes the minimal hardware and software requirements, the framework installation procedures, and the data collection and preprocessing stages. The time required for the following preparation steps heavily depends on the specifics of the devices. These procedures must be executed on each available machine, except if they have a shared file system.

The protocol below describes the specific steps for training a centralized and a federated multi-input NN for COVID-19 prognosis. The protocol can also be used with different data sources containing both images and tabular data; indeed, we have also used this protocol for Alzheimer’s disease detection.

### Requirements

Operating System: Ubuntu Linux 18.04+.

Python version: 3.8 (>=3.6, <3.9), recommended to use with Virtualenv.

Local memory: a minimum of 15 GB is required (dataset size is around 12 GB).

Deep Learning framework: Tensorflow 2+ or PyTorch 1.3+ (install the GPU version if you have available GPUs). Users can extend the list of supported Deep Learning frameworks if needed.

### Installing the OpenFL framework

**Timing: 15 min**

OpenFL is a framework-agnostic Python library for FL that enables organizations to collaboratively train a model without sharing sensitive information. OpenFL is a community-supported project, but it was originally developed by Intel Labs and Intel Internet of Things Group. Below are the required steps for installing OpenFL.

1. Open a new terminal window and create a new Virtualenv environment for the project. The recommended version of Python is 3.8 (>= 3.6, <3.9).

>python3.x -m venv "my_env"

2. Activate the virtual environment.

>source my_env/bin/activate

3. Install the OpenFL package from source.
  >python -m pip install -U pip setuptools wheel
  >cd openfl/
  >python -m pip install.
  a. Clone the OpenFL repository.
    >git clonehttps://github.com/intel/openfl.git.
  b. Install build tools, before installing OpenFL.
    If everything was done correctly, the >fx command in the virtual environment will confirm that OpenFL is installed.

### Data collection and preprocessing

**Timing: 25 min**

**Figure 1:**
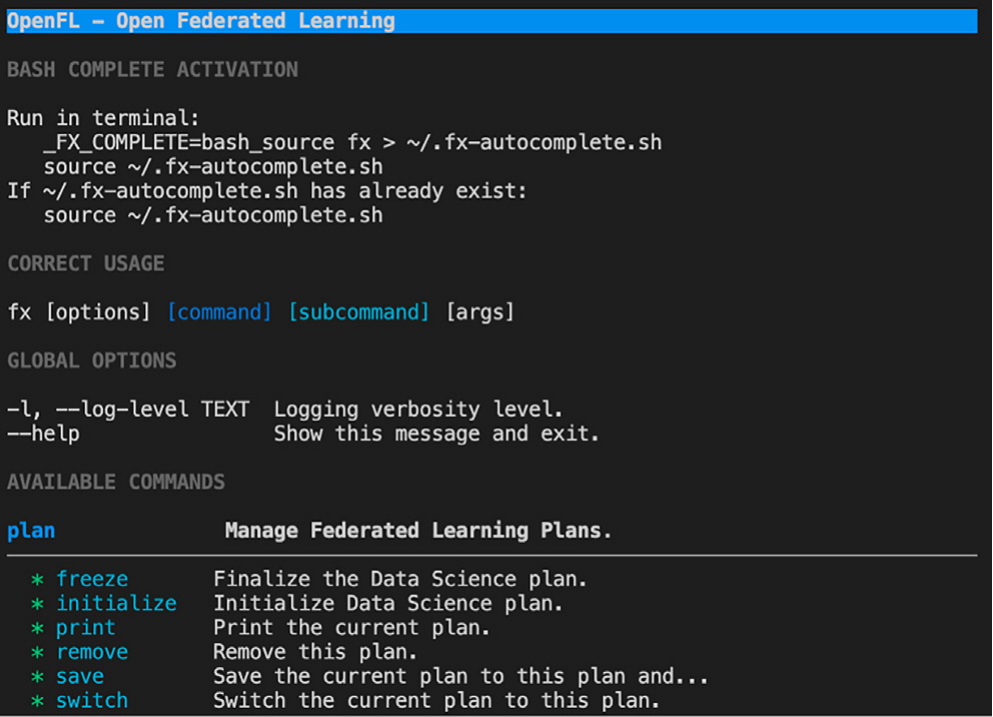
OpenFL working correctly

***Note:*** Your credentials will be sent by email in a few minutes.

***Note:*** The COVID-19 CXR dataset can be downloaded from https://aiforcovid.radiomica.it/(Figure 1).

**Figure 2:**
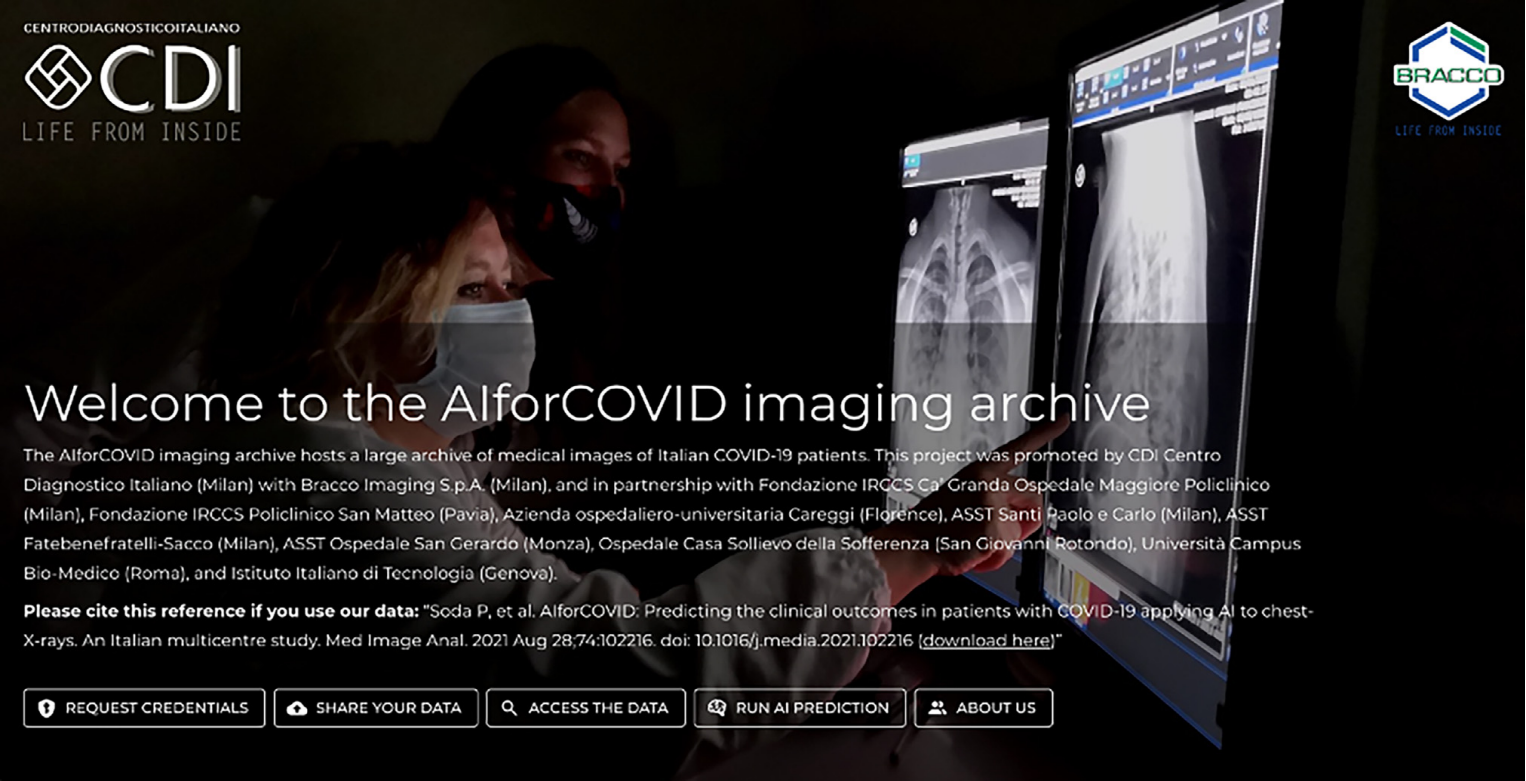
AIforCOVID imaging archive homepage

**Figure 3:**
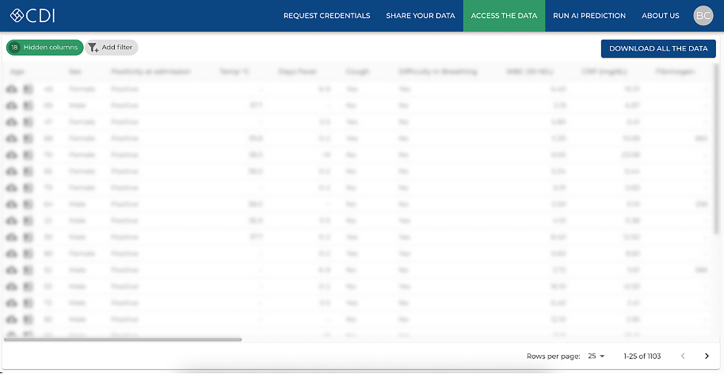
AIforCOVID data

***Note:*** If the provided link is not working, a dummy set of clinical data can be downloaded from the Supplemental ZIP files of this Protocol. X-ray images can be in any compatible format with PyTorch, such as .jpeg or .png.

4. Request credentials for accessing the data by clicking the “Request Credentials” button at the bottom left of the webpage.
  a. Insert your name, surname, institutional affiliation, and email, and accept the privacy conditions, the data user policy, and the Centro Diagnostico Italiano citation.

5. Click on the “Access the data” button present in https://aiforcovid.radiomica.it/and insert your credentials received.
6. After the login phase, you can finally download the data by clicking on the “Download all the data” button at the top right of the webpage (Figures 2 and 3).

At the end of the download process, you will have two Excel files containing the clinical parameters (i.e., age, sex, previous disease, …), and a zip file containing the chest X-ray scans. This dataset consists of data gathered across six different Italian hospitals during the first outbreak of COVID-19, for a total of 1589 patients, divided in 1103 for the training set, and 486 for the test set. For each subject of the dataset are provided sixteen clinical parameters (.xls format) and a chest X-rays image (.JPEG format). Below are described the data preprocessing stages:

7. Install the required packages (“requirements.yml” is provided in the GitHub repository of MERGE, see key resources table) in your virtualenv. Then, create a new Python script “preprocessing.py”, and import the required libraries.:

>import numpy as np

>import pandas as pd

>import matplotlib.pyplot as plt

>import seaborn as sns

>import os

>from sklearn.preprocessing import LabelEncoder

!pip install openpyxl

!pip install xlwt

import openpyxl

8. Put the datasets in the same folder of the Python script and import them. Replace the blank values with NaN. This step is necessary to make the dataset compatible with the next operations.

>dataset = pd.read_excel("trainClinData.xls")

>dataset = dataset.replace(r'ˆ\s∗$', np.nan, regex=True)

9. Encode target labels (the “Prognosis” column) with 0 and 1 values.

# Creating an instance of label Encoder.

>le = LabelEncoder()

# Using .fit_transform function to fit label encoder and return encoded label

>label = le.fit_transform(dataset['Prognosis'])

>dataset["Prognosis"] = label

10. Remove all the columns containing more than 500 missing values.

>del dataset["Fibrinogen"]

>del dataset["PCT"]

>del dataset["D_dimer"]

>del dataset["SaO2"]

>del dataset["Obesity"]

11. Repeat the 8–10 steps for the test set.

>testdataset = pd.read_excel("testClinData.xls")

>testdataset = testdataset.replace(r'ˆ\s∗$', np.nan, regex=True)

>le = LabelEncoder()

>label = le.fit_transform(testdataset['Prognosis'])

>testdataset["Prognosis"] = label

>del testdataset["Fibrinogen"]

>del testdataset["PCT"]

>del testdataset["D_dimer"]

>del testdataset["SaO2"]

>del testdataset["Obesity"]

12. The test set contains some columns with all NaN values hidden due to a data competition on this dataset. Indeed, these clinical parameters, i.e., the oxygen percentage, cardiovascular disease, ischemic heart disease, atrial fibrillation, heart failure, ictus, and position, have been removed because they are highly predictive features. The same features are present in the train set. However, they will not be considered for the NN training.
13. Save the resulting datasets.

>dataset.to_excel('trainClinData.xls', sheet_name='trainset')

>testdataset.to_excel('testClinData.xls',sheet_name='testset')

14. Open a terminal tab and navigate to the folder containing the Python script. Execute the script.

>python3 preprocessing.py

15. Merge the train and test clinical parameters in a single file named “trainANDtest.xls”. The train data encompass patients coming from six different hospitals, labeled from A to F, while the test data are collected from only one of those hospitals, the F one. In this way, the test set will contain patients coming from all the six hospitals. This step is fundamental for two reasons:
  a. In a centralized setting, it avoids overfitting the data coming from the hospital F.
  b. In a federated setting, it allows to respect the main FL principle: data never leave the local institution. Indeed, each client will train and test the model on data coming from a single hospital.

To merge the two datasets, copy and paste the rows of “trainClinData.xls” and “testClinData.xls” into a new file “trainANDtest.xls”.

After the clinical parameters preprocessing, we resize the images to 256 x 256.

16. In the same folder containing the clinical tabular and the zipped images, create a new Python script “resize.py”. Unzip the zip file containing the images. Inside there are two folders, “TrainSet” and “TestSet”.
17. Import the required libraries.

>import PIL

>from PIL import Image

>import os, sys

18. Instantiate necessary variables.

>path = "TrainSet/"

>dirs = os.listdir( path )

19. Create folders for saving the resized images.

# new folder path

>path_resized = 'Resized_Trainset' #the path where to save resized images

# create new folder

>if not os.path.exists(path_resized):

>  os.makedirs(path_resized)

# new folder path

>test_path_resized = 'Resized_Testset' #the path where to save resized images

# create new folder

>if not os.path.exists(test_path_resized):

> os.makedirs(test_path_resized)

20. Define the resize function.

>def resize():

 >for item in dirs:

  >if os.path.isfile(path+item):

   >im = PIL.Image.open(path+item)

   >f, e = os.path.splitext(path+item)

   >imResize = im.resize((256,256), Image.ANTIALIAS)

   >imResize.save('{}{}{}'.format(path_resized,'/',os.path.split(item)[1]))

21. Resize the train images.

>resize()

22. Resize the test images.

>path = "TestSet/"

>dirs = os.listdir( path )

>path_resized = test_path_resized

>resize()

23. Open a terminal tab and navigate to the folder containing the Python script. Execute the script.

>python3 resize.py

24. Put all the resized images from both the train and test set into a new folder “DATASET”.

The preprocessing steps are finished. At the end of this process, you need to have a directory containing the “trainANDtest.xls” file, and the “DATASET” folder containing the related chest X-rays scans.

## Key resources table

**Table:** 

REAGENT or RESOURCE	SOURCE	IDENTIFIER
**Deposited data**		

COVID-19 chest X-rays (AIforCOVID)	Soda et al.^2^	https://aiforcovid.radiomica.it/
Dummy COVID-19 clinical parameters		https://doi.org/10.17632/ktyksxb5nr.1

**Software and algorithms**		

OpenFL	Foley et al.^3^	https://github.com/securefederatedai/openfl/tree/develop
MERGE: a model for multi-input biomedical federated learning	Casella et al.^1^	https://github.com/CasellaJr/Multi-Input-Neural-Networks-in-Federated-Learning/tree/v1.0.0 https://doi.org/10.5281/zenodo.8218953

**Others**		

CPU	Intel	Xeon processor (Skylake)
GPU	NVIDIA	Tesla T4

## Step-by-step method details

Herein, we describe Step-by-step methods for training MERGE, a federated multi-input NN for COVID-19 prognosis. Before showing the steps for training a federated model, we describe the stages for a centralized scenario in which the data are collected in a single data lake. To illustrate these various steps, we use, as an example, the training of a multi-input NN in both a centralized and federated version.

### Centralized training

**Timing: 12 h**

The time required for the centralized training highly depends on GPUs availability. Indicated timing is the approximation of the time required for training on a single NVIDIA Tesla T4.

1. Create a new Python script “centralized_multi_input.py” in the same directory containing the datasets. Import the required libraries.

>import torch.nn.functional as F

>import torch

>import torchvision

>from matplotlib import pyplot as plt

>from torchvision import utils

>import numpy as np

>import torch.nn as nn

>import os

>import pandas as pd

>import PIL

>from PIL import Image

>from torch.utils.data import Dataset, DataLoader, random_split

>from torchvision import transforms

>from sklearn.model_selection import StratifiedKFold

>from sklearn.model_selection import KFold

>from torch.utils.tensorboard import SummaryWriter

>from sklearn.metrics import accuracy_score, precision_score, >recall_score, f1_score, confusion_matrix

>import time

>import pickle

2. Import the tabular data and remove unnecessary columns.

***Note:*** the “type_hospital” variable refers to the type of data we are training on (i.e., images, tabular, or multi-input) and from which hospital they come. In this example, we consider a multi-input NN training on data coming from all the hospitals and collected in a single data lake. Moreover, to select only data coming from a specific hospital, uncomment the last line and change X to 1,…,6 according to the hospital you want.

>type_hospital = “multi_ALL”

>data_path = "./"

>images = os.listdir(f"{data_path}DATASET")

>df = pd.read_excel("trainANDtest.xls")

>del df["Row_number"]

>del df["Unnamed: 0"]

>#df = df[df["Hospital"]==X] #change X to 1,…,6 if you want to use only data coming from one of the six hospitals.

3. The following lines of code import the required libraries, check if GPUs are available, and, if yes, set a deterministic seed for GPU reproducibility; otherwise, the numpy seed will be used for CPU reproducibility.

>save_folder = "MODELS"

>myseed = 0

>torch.manual_seed(myseed)

>np.random.seed(myseed)

>generator=torch.Generator()

>generator.manual_seed(myseed)

>%matplotlib inline

>dev = torch.device("cuda" if torch.cuda.is_available() else "cpu")

>torch.backends.cudnn.deterministic = True

>torch.backends.cudnn.benchmark = False

4. For this setting, a test set representative of the distribution of all the hospitals can be downloaded at the following link: https://github.com/CasellaJr/Multi-Input-Neural-Networks-in-Federated-Learning/blob/main/COVID/globalTEST.xls.

>globalTEST = pd.read_excel("globalTEST.xls")

>del globalTEST["Row_number"]

>del globalTEST["Unnamed: 0"]

>globalTEST1 = globalTEST[globalTEST["Hospital"]==1]

>globalTEST2 = globalTEST[globalTEST["Hospital"]==2]

>globalTEST3 = globalTEST[globalTEST["Hospital"]==3]

>globalTEST4 = globalTEST[globalTEST["Hospital"]==4]

>globalTEST5 = globalTEST[globalTEST["Hospital"]==5]

>globalTEST6 = globalTEST[globalTEST["Hospital"]==6]

5. Create a PyTorch Custom Dataset class.

***Note:*** This class will return an image, tabular, and label associated with a patient. For considering just images or tabular separately, remove one of them from the return statement according to which type of input source you want to use.

>class ImageDataset(Dataset):

 """Tabular and Image dataset."""

 >def __init__(self, indices, image_dir, transform=None):

  >self.image_dir = image_dir

  >self.indices = indices

  >self.transform = transform

 >def __len__(self):

  >return len(self.indices)

 >def __getitem__(self, idx):

  >if torch.is_tensor(idx):

   >idx = idx.tolist()

  >tabular = self.indices.iloc[idx, 0:]

  >y = tabular["Prognosis"]

  >image = PIL.Image.open(f"{self.image_dir}/{tabular['ImageFile']}")

  >image = image.convert('L')

  >image = np.array(image)

  >#image = image[..., :3]

  >image = transforms.functional.to_tensor(image)

  >tabular = tabular[['Age', 'Sex', 'PositivityAtAdmission',

  'Temp_C', 'DaysFever', 'Cough', 'DifficultyInBreathing', 'WBC', 'RBC',

  'CRP', 'Glucose', 'LDH', 'INR', 'PaO2', 'PaCO2', 'pH',

  'HighBloodPressure', 'Diabetes', 'Dementia', 'BPCO', 'Cancer',

  'ChronicKidneyDisease', 'RespiratoryFailure']]

  >tabular = tabular.tolist()

  >tabular = torch.FloatTensor(tabular)

  >if self.transform:

   >image = self.transform(image)

  >return image, tabular, y

6. Define data augmentation stages and split data in train and test. The train set will be divided into train and validation later.

>from torchvision import transforms as T

>my_transform = T.Compose([T.Resize((256,256)),

        T.RandomApply(

         [T.RandomHorizontalFlip(),

          T.RandomCrop(256, padding=4)],

         p=.5

        )])

>from sklearn.model_selection import train_test_split

>tv_idx, test_idx = train_test_split(np.arange(len(df["Prognosis"])), test_size=0.2, shuffle=True, stratify=df["Prognosis"])

>train_val_df = df.iloc[tv_idx]

>train_val = ImageDataset(indices=train_val_df, image_dir=f"{data_path}DATASET", transform=my_transform)

>test_df = df.iloc[test_idx]

>test_set = ImageDataset(indices=test_df, image_dir=f"{data_path}DATASET", transform=my_transform)

7. Extract test data for each hospital from the global test set.

>global_test_set = ImageDataset(indices=globalTEST, image_dir=f"{data_path}DATASET", transform=my_transform)

>global_test_set1 = ImageDataset(indices=globalTEST1, image_dir=f"{data_path}DATASET", transform=my_transform)

>global_test_set2 = ImageDataset(indices=globalTEST2, image_dir=f"{data_path}DATASET", transform=my_transform)

>global_test_set3 = ImageDataset(indices=globalTEST3, image_dir=f"{data_path}DATASET", transform=my_transform)

>global_test_set4 = ImageDataset(indices=globalTEST4, image_dir=f"{data_path}DATASET", transform=my_transform)

>global_test_set5 = ImageDataset(indices=globalTEST5, image_dir=f"{data_path}DATASET", transform=my_transform)

>global_test_set6 = ImageDataset(indices=globalTEST6, image_dir=f"{data_path}DATASET", transform=my_transform)

8. Define the BasicBlock class need for our Convolutional Neural Network.

>class BasicBlock(nn.Module):

 >expansion = 1

 >def __init__(self, in_planes, planes, stride=1):

  >super(BasicBlock, self).__init__()

  >self.conv1 = nn.Conv2d(

   in_planes, planes, kernel_size=3, stride=stride, padding=1, bias=False)

  >self.bn1 = nn.BatchNorm2d(planes)

  >self.conv2 = nn.Conv2d(planes, planes, kernel_size=3,

  stride=1, padding=1, bias=False)

  >self.bn2 = nn.BatchNorm2d(planes)

  >self.shortcut = nn.Sequential()

  >if stride != 1 or in_planes != self.expansion∗planes:

   >self.shortcut = nn.Sequential(

    nn.Conv2d(in_planes, self.expansion∗planes,

        kernel_size=1, stride=stride, bias=False),

    >nn.BatchNorm2d(self.expansion∗planes)

   )

  >def forward(self, x):

   >out = F.relu(self.bn1(self.conv1(x)))

   >out = self.bn2(self.conv2(out))

   >out += self.shortcut(x)

   >out = F.relu(out)

   >return out

9. Define the “ResNet” class. It can be used to instantiate every version of ResNet (i.e., ResNet-18, ResNet-54, …). Our “ResNet” class allows for creating a multi-input NN, where the convolutional part is equal to a ResNet, but it takes as input both images and tabular.

>class ResNet(nn.Module):

 >def __init__(self, block, num_blocks, in_channels=1, num_classes=2):

  >super(ResNet, self).__init__()

  >torch.manual_seed(myseed)

  >self.in_planes = 64

  >self.conv1 = nn.Conv2d(in_channels, 64, kernel_size=3,

  stride=1, padding=1, bias=False)

  >self.bn1 = nn.BatchNorm2d(64)

  >self.layer1 = self._make_layer(block, 64, num_blocks[0], stride=1)

  >self.layer2 = self._make_layer(block, 128, num_blocks[1], stride=2)

  >self.layer3 = self._make_layer(block, 256, num_blocks[2], stride=2)

  >self.layer4 = self._make_layer(block, 512, num_blocks[3], stride=2)

  >self.avgpool = nn.AdaptiveAvgPool2d((1, 1))

  >self.linear = nn.Linear(512 ∗ block.expansion, 10)

  >self.relu = nn.ReLU()

  >self.ln1 = nn.Linear(23, 50)

  >self.ln2 = nn.Linear(50, 50)

  >self.ln3 = nn.Linear(50, 10)

  >self.ln4 = nn.Linear(20, 1)

 >def _make_layer(self, block, planes, num_blocks, stride):

  >strides = [stride] + [1]∗(num_blocks-1)

  >layers = []

  >for stride in strides:

   >layers.append(block(self.in_planes, planes, stride))

   >self.in_planes = planes ∗ block.expansion

  >return nn.Sequential(∗layers)

 >def forward(self, x, tab):

  >out = F.relu(self.bn1(self.conv1(x)))

  >out = self.layer1(out)

  >out = self.layer2(out)

  >out = self.layer3(out)

  >out = self.layer4(out)

  >out = self.avgpool(out)

  >out = out.view(out.size(0), -1)

  >out = self.linear(out)

  >out = self.relu(out)

  >tab = self.ln1(tab)

  >tab = self.relu(tab)

  >tab = self.ln2(tab)

  >tab = self.relu(tab)

  >tab = self.ln3(tab)

  >tab = self.relu(tab)

  >out = torch.cat((out, tab), dim=1)

  >out = self.relu(out)

  >out = self.ln4(out)

  >return out

def ResNet18(in_channels, num_classes):

return ResNet(BasicBlock, [2, 2, 2, 2], in_channels=in_channels, num_classes=num_classes)

model = ResNet18(1,1)

print(model)

10. Define optimizer, loss function and scheduler.

# Define an optimizer

import torch.optim as optim

optimizer = optim.Adam(model.parameters(), lr = 0.0001)

# Define a loss

criterion = nn.BCEWithLogitsLoss()

scheduler = torch.optim.lr_scheduler.OneCycleLR(optimizer, max_lr = 0.01, epochs=1, steps_per_epoch=350)

11. Define the evaluation function.

>def eval_model(model, data_loader):

  >model = model.to(dev)

  >model.eval() # Set model to eval mode

  >true_preds, num_preds = 0., 0.

  >with torch.no_grad(): # Deactivate gradients for the following code

   >for data_inputs, tabular, data_labels in data_loader:

    >data_inputs = data_inputs.to(dev)

    >tabular = tabular.to(dev)

    >data_labels = data_labels.to(dev)

    # Determine prediction of model on dev set

    >preds = model(data_inputs, tabular)

    >preds = preds.double()

    >data_labels = data_labels.unsqueeze(1)

    >data_labels = data_labels.float()

    >pred_labels = (preds >= 0).long() # Binarize predictions to 0 and 1

    # Keep records of predictions for the accuracy metric (true_preds=TP+TN, num_preds=TP+TN+FP+FN)

    >true_preds += (pred_labels == data_labels).sum()

    >num_preds += data_labels.shape[0]

  >acc = true_preds / num_preds

  >acc = acc.cpu().numpy()

  >return acc

12. Define the training function. As it is too long, it can be downloaded from the following link: https://pastebin.com/xpFMDE3V. It is also available on the MERGE repository.
13. Create a function for resetting weights. It will be used after the training on each stratified fold split (5 splits in total).

>def reset_weights(m):

 >if isinstance(m, nn.Conv2d) or isinstance(m, nn.Linear):

  >m.reset_parameters()

14. Define test data loaders.

>test_loader = DataLoader(test_set, batch_size=8, num_workers=2, drop_last=False, shuffle=False, generator=generator)

>global_test_loader = DataLoader(global_test_set, batch_size=8, num_workers=2, drop_last=False, shuffle=False, generator=generator)

>global_test_loader1 = DataLoader(global_test_set1, batch_size=8, num_workers=2, drop_last=False, shuffle=False, generator=generator)

>global_test_loader2 = DataLoader(global_test_set2, batch_size=8, num_workers=2, drop_last=False, shuffle=False, generator=generator)

>global_test_loader3 = DataLoader(global_test_set3, batch_size=8, num_workers=2, drop_last=False, shuffle=False, generator=generator)

>global_test_loader4 = DataLoader(global_test_set4, batch_size=8, num_workers=2, drop_last=False, shuffle=False, generator=generator)

>global_test_loader5 = DataLoader(global_test_set5, batch_size=8, num_workers=2, drop_last=False, shuffle=False, generator=generator)

>global_test_loader6 = DataLoader(global_test_set6, batch_size=8, num_workers=2, drop_last=False, shuffle=False, generator=generator)

15. Create 5 stratified folds and start training.

>skf = StratifiedKFold(n_splits=5)

>epochs=100

>for fold,(train_idx, val_idx) in enumerate(skf.split(train_val, tv_labels)):

 >writer = SummaryWriter("runs/CON F1 PRECISION RECALL/MULTI/HOSPITAL B", filename_suffix=f"_F{fold}_E{epochs}_")

 >print('------------fold no---------{}----------------------'.format(fold))

 >train_df = df.iloc[train_idx]

 >train_set = ImageDataset(indices=train_df, image_dir=f"{data_path}DATASET", transform=my_transform)

 >val_df = df.iloc[val_idx]

 >val_set = ImageDataset(indices=val_df, image_dir=f"{data_path}DATASET", transform=my_transform)

 >train_loader = DataLoader(train_set, batch_size=8, num_workers=2, drop_last=True, shuffle=True, generator=generator)

 >val_loader = DataLoader(val_set, batch_size=8, num_workers=2, drop_last=False, shuffle=False, generator=generator)

# Define dictionary of loaders

 >loaders = {"train": train_loader,

    "val": val_loader,

    "test": test_loader}

# Start training

 >acc_glob, acc_1, acc_2, acc_3, acc_4, acc_5, acc_6, preds_and_labels = train(model, loaders, optimizer, criterion, epochs=100, dev=dev, model_name=f"{fold}_multi-input")

>model.apply(reset_weights)

16. Keep trace of the results with Tensorboard.

***Note:*** this step is not mandatory, but it is helpful for tracking metrics. Metrics tracking is also possible thanks to the training function. Indeed, all the metrics will be reported for every single epoch. Moreover, at the end of training, two plots tracking losses and accuracies for each train/validation/test split will be generated.

>writer.add_scalar("Global test set accuracy", acc_glob, fold)

>writer.add_scalar("Global test set 1 accuracy", acc_1, fold)

>writer.add_scalar("Global test set 2 accuracy", acc_2, fold)

>writer.add_scalar("Global test set 3 accuracy", acc_3, fold)

>writer.add_scalar("Global test set 4 accuracy", acc_4, fold)

>writer.add_scalar("Global test set 5 accuracy", acc_5, fold)

>writer.add_scalar("Global test set 6 accuracy", acc_6, fold)

>writer.flush()

writer.close()

17. Open a new terminal tab in the same directory and run “python centralized_multi_input.py”.

This section described the steps necessary for training a centralized multi-input model on the COVID-19 chest X-rays dataset. Metrics will be saved in a “runs” directory. To analyze them, open a new terminal tab in the same directory of the script and run “tensorboard –logdir = runs”.

### Federated training

**Timing: 12 h**

As for the previous scenario, the time required for the federated training highly depends on GPUs availability.

The following steps describe how to train MERGE, a model for multi-input biomedical federated learning. The federation will encompass six Collaborators, i.e., clients (hospitals) holding local data, and one Aggregator, i.e., the server that aggregates the models. Each Collaborator will train a local model on data coming only from one of the six hospitals (A to F).

18. In the same directory containing the data, create three folders respectively named “director”, “envoy” and “workspace”.
19. Navigate to the “director”. The Director is responsible for the creation and management of an Aggregator. Create a human-readable data serialization file (“director_config.yaml”) and a bash script (“start_director.sh”).
  a. “director_config.yaml” is a configuration file describing the listening host (localhost), the listen port, the sample, and the target shape of the Director.
    settings:
     listen_host: 0.0.0.0
     listen_port: 50051
     sample_shape: ['1', '256', '256']
     target_shape: ['1', '256', '256']
  b. “start_director.sh” will help us in running the Director. The Director is a long-lived OpenFL entity and is the central node of the federation. It accepts connections from Envoys, the OpenFL clients. The Director coordinates the aggregation process and it is responsible for sending tasks and data to the envoys. The code below starts a Director entity without encrypting the communication network (by disabling Transport Layer Security. For more information about TLS, check the official OpenFL documentation) and according to the parameters of the “director_config.yaml”. In particular, it will execute a Director service on localhost, with port 50051 and it will accept envoys compliant with the expected sample and target shapes.
    #!/bin/bash
    set -e
    fx director start --disable-tls -c director_config.yaml
20. Navigate to the “envoy” folder. Create a Python script (“covid_shard_descriptor.py”), a YAML (“envoy_configX.yaml”), and a bash script (“start_envoyX.sh”) for each client of the federation, where X represents the number associated with that client. Considering that we have six clients, this directory will contain 13 files: one shard descriptor, six configuration files, and six bash scripts.
  a. The “covid_shard_descriptor.py” is responsible for sharding the dataset. In particular, working in synergy with the YAML configuration files it will assign the right data to the various clients.
    i. First of all, import the required libraries.
      >import logging
      >import os
      >from typing import List
      >from torch.utils.data import Dataset, random_split
      >import pandas as pd
      >from sklearn.model_selection import train_test_split
      >import torch
      >import PIL
      >from PIL import Image
      >from torchvision import transforms as T
      >import numpy as np
      >import requests
      >from openfl.interface.interactive_api.shard_descriptor >import ShardDataset
      >from openfl.interface.interactive_api.shard_descriptor >import ShardDescriptor
      >logger = logging.getLogger(__name__)
    ii. Create the CovidShardDataset Class.
      >class CovidShardDataset(ShardDataset):
      > """Covid Shard dataset class."""
      > def __init__(self, img, tab, y, data_type, rank=1, worldsize=1):
      >   """Initialize CovidDataset."""
      >   self.data_type = data_type
      >   self.rank = rank
      >   self.worldsize = worldsize
      >   self.img = img[self.rank - 1::self.worldsize]
      >   self.tab = tab[self.rank - 1::self.worldsize]
      >   self.y = y[self.rank - 1::self.worldsize]
      >  def __getitem__(self, index: int):
      >   """Return an item by the index."""
      >   return self.img[index], self.tab[index], self.y[index]
      >  def __len__(self):
      >   """Return the len of the dataset."""
      >   return len(self.img)
    iii. Create the CovidShardDescriptor Class.
      >class CovidShardDescriptor(ShardDescriptor):
      >  """Covid Shard descriptor class."""
      >  def __init__(
      >    self,
      >    rank_worldsize: str = '1, 1',
      >    hospital: str = '',
      >    ∗∗kwargs
      >  ):
      >    """Initialize CovidShardDescriptor."""
      >    self.rank, self.worldsize = tuple(int(num) for num in rank_worldsize.split(','))
      >    self.hospital = hospital
      >    (img_train, tab_train, y_train), (img_test, tab_test, y_test) = self.download_data()
      >    self.data_by_type = {
      >     'train': (img_train, tab_train, y_train),
      >     'val': (img_test, tab_test, y_test)
      >   }
      >  def get_shard_dataset_types(self) -> List[str]:
      >   """Get available shard dataset types."""
      >  return list(self.data_by_type)
      >  def get_dataset(self, dataset_type='train'):
      >   """Return a shard dataset by type."""
      >   if dataset_type not in self.data_by_type:
      >    raise Exception(f'Wrong dataset type: {dataset_type}')
      >   return CovidShardDataset(
      >    ∗self.data_by_type[dataset_type],
      >    data_type=dataset_type,
      >    rank=self.rank,
      >    worldsize=self.worldsize
    iv. The CovidShardDescriptor Class holds the following properties.
      >@property
      >def sample_shape(self):
      >  return ['1', '256', '256']
      >@property
      >def target_shape(self):
      >  return ['1', '256', '256']
      >@property
      >def dataset_description(self) -> str:
      >  return (f'Images-tabular dataset, shard number {self.rank}'
      >  f' out of {self.worldsize}')
    v. The CovidShardDescriptor Class implements the “download_data” function, which is responsible for splitting and returning the data in the correct format. This function calls the ImageDataset Class, which is the same as previous described for the centralized scenario (bullet point 5)
      >def download_data(self):
      >  """Download prepared dataset."""
      >  img_train = []
      >  tab_train = []
      >  y_train = []
      >  img_test = []
      >  tab_test = []
      >  y_test = []
      >  my_transform = T.Compose([T.Resize((256,256)),
      >          T.RandomApply(
      > [T.RandomHorizontalFlip(),
      >          T.RandomCrop(256, padding=4)],
      >         p=.5
      >        )])
      >  image_data = ImageDataset(ospedale = self.hospital, excel_file="../trainANDtest.xls", image_dir="../DATASET", transform=my_transform)
      >  train_size = int(0.80 ∗ len(image_data))
      >  val_size = int((len(image_data) - train_size))
      >  image_data, test_image_data = random_split(image_data, (train_size, val_size))
      >  for item in image_data:
      >   img_train.append(item[0])
      >   tab_train.append(item[1])
      >   y_train.append(item[2])
      >
      >  for item in test_image_data:
      >   img_test.append(item[0])
      >   tab_test.append(item[1])
      >   y_test.append(item[2])
      >  return (img_train, tab_train, y_train), (img_test, tab_test, y_test)
21. The “envoy_configX.yaml” is a configuration file describing the specifics of the Envoy. Assign to X the value of each hospital (1–6).

params:

 cuda_devices: []

optional_plugin_components: {}

shard_descriptor:

 template: covid_shard_descriptor.CovidShardDescriptor

 params:

  data_folder: covid_data

  rank_worldsize: 1,1

  hospital: X

22. “start_envoyX.sh” will help us in running the Envoys. For this example, we are considering a simulated federation scenario. If you want a real federation, change “localhost” with the Fully Qualified Domain Name (FQDN) of your devices.
  #!/bin/bash
  set -e
  fx envoy start -n env_X --disable-tls --envoy-config-path envoy_configX.yaml -dh localhost -dp 50051
  a. To find your FQDN prompt:
    >hostname --fqdn
23. Create and connect to the federation by running the bash scripts just created. Start from the director script, and only once it is active, connect the envoys.
24. Navigate to the “workspace” directory and create a new Python script “federated_multi_input.py” in the same directory containing the datasets. Import the required libraries. In the following snippet of code, the variable “myseed” is responsible for reproducibility purposes. MERGE^1^ ran this protocol five times, changing this variable with values from 0 to 4 and averaging the results.

>import os

>import glob

>from PIL import Image

>import numpy as np

>import torch

>import torch.nn as nn

>import torch.nn.functional as F

>import torch.optim as optim

>from openfl.interface.interactive_api.federation import Federation

>from openfl.interface.interactive_api.experiment import TaskInterface, DataInterface, ModelInterface, FLExperiment

>from copy import deepcopy

>import torchvision

>from torchvision import transforms as T

>from torch.utils.data import Dataset

>from torch.utils.data import DataLoader

>import tqdm

>myseed = 0

>torch.manual_seed(myseed)

>np.random.seed(myseed)

>torch.backends.cudnn.deterministic = True

>torch.backends.cudnn.benchmark = False

25. Connect to the federation.

>client_id = 'api'

>cert_dir = 'cert'

>director_node_fqdn = 'localhost'

>federation = Federation(client_id=client_id, director_node_fqdn=director_node_fqdn, director_port='50051', tls=False)

***Optional:*** Request info about sample and target shapes and double-check that all the clients are connected to the federation.

federation.target_shape

shard_registry = federation.get_shard_registry()

print(shard_registry)

dummy_shard_desc = federation.get_dummy_shard_descriptor(size=10)

dummy_shard_dataset = dummy_shard_desc.get_dataset('train')

sample, target = dummy_shard_dataset[0]

print(sample.shape)

print(target.shape)

26. Create a Dataset PyTorch class.

>class TransformedDataset(Dataset):

> def __init__(self, dataset, transform=None, target_transform=None):

>  """Initialize Dataset."""

>  self.dataset = dataset

> def __len__(self):

>  """Length of dataset."""

>  return len(self.dataset)

> def __getitem__(self, index):

>  img, tab, label = self.dataset[index]

> return img, tab, label

27. Use the previous class to federate the COVID-19 dataset.

>class COVIDDataset(DataInterface):

> def __init__(self, ∗∗kwargs):

>  self.kwargs = kwargs

> @property

> def shard_descriptor(self):

>  return self._shard_descriptor

> @shard_descriptor.setter

> def shard_descriptor(self, shard_descriptor):

> self._shard_descriptor = shard_descriptor

>

>  self.train_set = TransformedDataset(

>  self._shard_descriptor.get_dataset('train'),

>  )

>  self.valid_set = TransformedDataset(

>  self._shard_descriptor.get_dataset('val'),

> )

> def get_train_loader(self, ∗∗kwargs):

>  generator=torch.Generator()

>  generator.manual_seed(myseed)

>   return DataLoader(

>    self.train_set, batch_size=self.kwargs['train_bs'], shuffle=True, generator=generator

>    )

> def get_valid_loader(self, ∗∗kwargs):

>  return DataLoader(self.valid_set, batch_size=self.kwargs['valid_bs'])>

> def get_train_data_size(self):

>  return len(self.train_set)>

> def get_valid_data_size(self):

>  return len(self.valid_set)

> fed_dataset = COVIDDataset(train_bs=8, valid_bs=8)

28. Instantiate the same multi-input model described before for the centralized scenario (bullet point 9).

>model_net = ResNet18(1,1)

29. Define the optimizer and the training criterion.

>params_to_update = []

>for param in model_net.parameters():

> if param.requires_grad == True:

>  params_to_update.append(param)

>optimizer = optim.Adam(params_to_update, lr = 0.0001)

>criterion = nn.BCEWithLogitsLoss()

>def cross_entropy(output, target):

> criterion = nn.BCEWithLogitsLoss()

> loss = criterion(output, target)

> return loss

30. Register the model for the OpenFL framework, as well as the FL tasks (local model training, local model validation, and global model validation).

>framework_adapter = 'openfl.plugins.frameworks_adapters.pytorch_adapter.FrameworkAdapterPlugin'

>model_interface = ModelInterface(model=model_net, optimizer=optimizer, framework_plugin=framework_adapter)

>initial_model = deepcopy(model_net)

>import pickle

>from sklearn.metrics import accuracy_score, precision_score, recall_score, f1_score, confusion_matrix

>task_interface = TaskInterface()

>train_custom_params={'precision_score': precision_score,

> 'recall_score': recall_score, 'f1_score': f1_score,

>      }

>def function_defined_in_notebook(some_parameter):

> print(f'Also I accept a parameter and it is {some_parameter}')

>@task_interface.register_fl_task(model='net_model', data_loader='train_loader', \

> device='device', optimizer='optimizer')

>@task_interface.add_kwargs(∗∗train_custom_params)

>@task_interface.add_kwargs(∗∗{'some_parameter': 42})

31. Define a training function.

>def train(net_model, train_loader, optimizer, device, precision_score, recall_score, f1_score,

>   loss_fn=cross_entropy, some_parameter = None):

> torch.manual_seed(myseed)

> device='cuda'

> function_defined_in_notebook(some_parameter)

> train_loader = tqdm.tqdm(train_loader, desc="train")

> net_model.train()

> net_model.to(device)

> losses = []

> total_acc, sum_precision, sum_recall, sum_f1 = 0,0,0,0

> epochs = 1

> for epoch in range(epochs):

>  for img, tabular, target in train_loader:

>   img, tabular, target = torch.tensor(img).to(device), torch.tensor(tabular).to(device), torch.tensor(target).to(device, dtype=torch.int64)

>   optimizer.zero_grad()

>   output = torch.flatten(net_model(img, tabular)) #multi input

>   output = output.float()

>   loss = criterion(output, target.float())

>   loss.backward()

>   optimizer.step()

>   losses.append(loss.detach().cpu().numpy())

>   pred_labels = (output >= 0).float() # Binarize predictions to 0 and 1

>   batch_accuracy = (pred_labels == target).sum().item()/tabular.size(0)

>   total_acc += batch_accuracy

>   sum_precision += precision_score(target.cpu().numpy(), pred_labels.cpu().numpy(), average='binary', zero_division=0)

>   sum_recall += recall_score(target.cpu().numpy(), pred_labels.cpu().numpy(), average='binary', zero_division=0)

>   sum_f1 += f1_score(target.cpu().numpy(), pred_labels.cpu().numpy(), average='binary', zero_division=0)

> return {'train_loss': np.mean(losses),

>   'train_acc': total_acc/len(train_loader),

>   'train_prec': sum_precision/len(train_loader),

>   'train_rec': sum_recall/len(train_loader),

>   'train_f1': sum_f1/len(train_loader)

>   }

32. Define the validation function.

>val_custom_params={ 'precision_score': precision_score,

>     'recall_score': recall_score, 'f1_score': f1_score,

>     }

>@task_interface.register_fl_task(model='net_model', data_loader='val_loader', device='device')

>@task_interface.add_kwargs(∗∗val_custom_params)

>def validate(net_model, val_loader, device, precision_score, recall_score, f1_score):

> torch.manual_seed(myseed)

> device = torch.device('cuda')

> net_model.eval()

> net_model.to(device)

> losses = []

> total_acc, sum_precision, sum_recall, sum_f1 = 0,0,0,0

> val_loader = tqdm.tqdm(val_loader, desc="validate")

> val_score = 0

> total_samples = 0

> with torch.no_grad():

> for img, tabular, target in val_loader:

>  #print("TARGET VAL LOADER:", target)

>  samples = target.shape[0]

>  total_samples += samples

>  img, tabular, target = torch.tensor(img).to(device), torch.tensor(tabular).to(device), torch.tensor(target).to(device, dtype=torch.int64)

>  output = torch.flatten(net_model(img, tabular)) #multi input

>  output = (output >= 0.0).float() #binarize predictions

>  loss = criterion(output, target.float())

>  losses.append(loss.detach().cpu().numpy())

>  batch_accuracy = (output == target).sum().item()/tabular.size(0)

>  total_acc += batch_accuracy

>  sum_precision += precision_score(target.cpu().numpy(), output.cpu().numpy(), average='binary', zero_division=0)

>  sum_recall += recall_score(target.cpu().numpy(), output.cpu().numpy(), average='binary', zero_division=0)

>  sum_f1 += f1_score(target.cpu().numpy(), output.cpu().numpy(), average='binary', zero_division=0)

>  val_score += output.eq(target).sum().cpu().numpy()

> return {'val_loss': np.mean(losses),

>   'acc': val_score / total_samples,

>   'val_prec': sum_precision/len(val_loader),

>   'val_rec': sum_recall/len(val_loader),

>   'val_f1': sum_f1/len(val_loader)

>   }

33. Start the FL experiment and stream the metrics.

>experiment_name = 'POSTREVIEWS_federated_covid_MULTI_6hospitals'

>fl_experiment = FLExperiment(federation=federation, experiment_name=experiment_name)

>fl_experiment.start(

> model_provider=model_interface,

> task_keeper=task_interface,

> data_loader=fed_dataset,

> rounds_to_train=100,

> opt_treatment='CONTINUE_GLOBAL'

>)

>fl_experiment.stream_metrics(tensorboard_logs=True)

34. Run the “federated_multi_input.py” script. Terminal will show the model’s metrics, that will also be recorded as TensorBoard events, and saved in a “runs” directory.

## Expected outcomes

**Figure 4:**
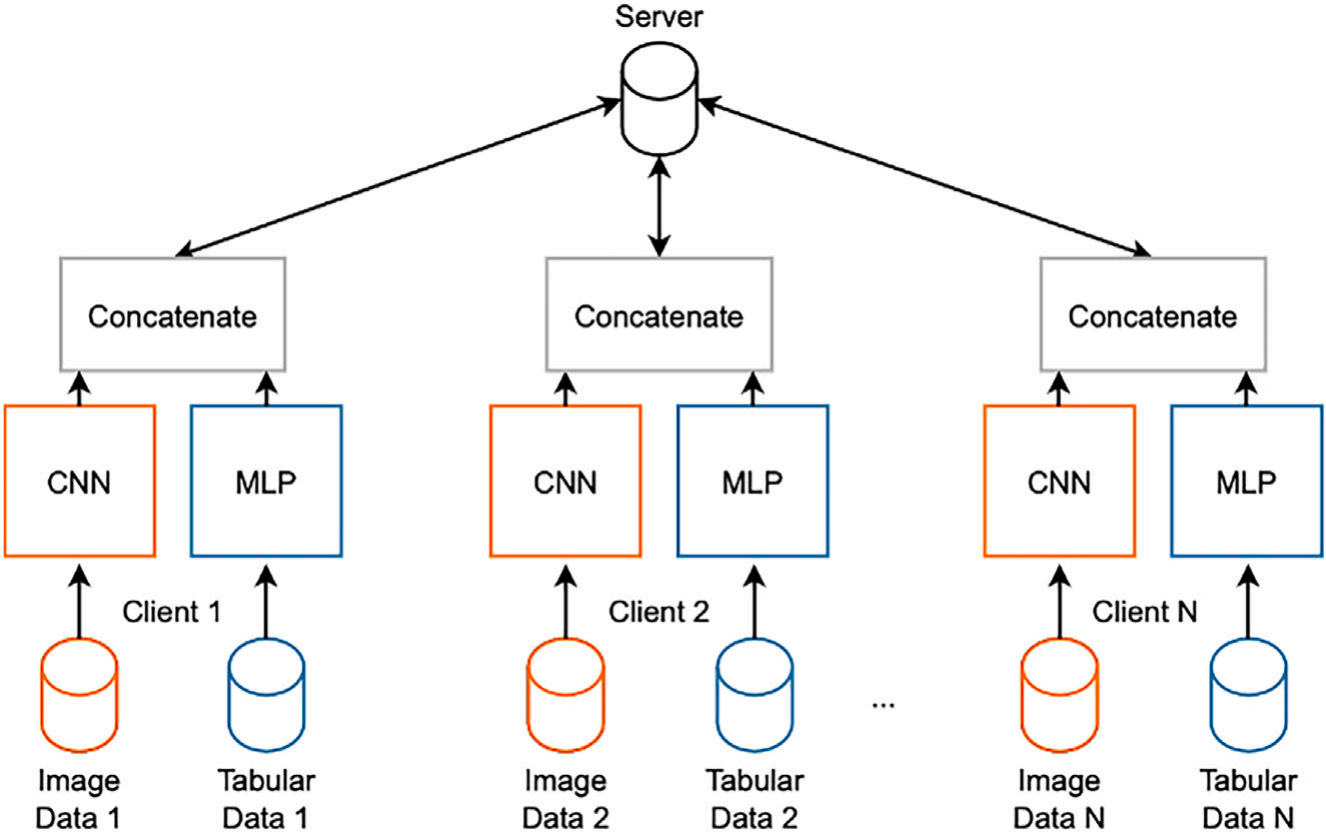
Federated learning with multi-input neural networks

**Figure 5:**
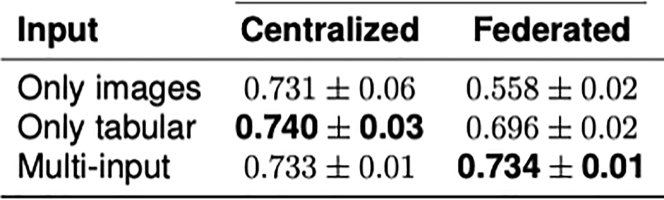
Accuracies of MERGE

**Figure 6:**
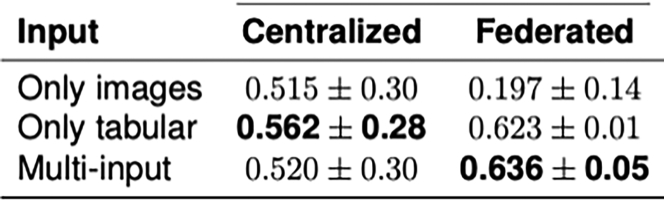
F1-scores of MERGE

MERGE introduces an FL setting with the advantage of leveraging multiple input sources for solving classification tasks in the bio-medical environment in a privacy-compliant way. The basic assumption for this approach is that each federation participant has both data types, images, and tabular, locally available and accessible (Figure 4). The goodness of this protocol has been demonstrated by running several tests based on images combined with tabular data from the COVID-19 chest X-rays dataset. However, this protocol has also been tested for the Alzheimer’s disease detection by training on the ADNI study. MERGE has been compared with models trained only on images or tabular. Results show that enabling multi-input architectures in the FL framework allows for improving the performance regarding both accuracy and f1-score with respect to non-federated models while complying with data protection practices. If the steps presented in this protocol are executed successfully, the results will be the same as those of MERGE (Figures 5 and 6).^1^

## Limitations

The main objective of MERGE^1^ was to demonstrate the feasibility of a horizontal federated multi-input architecture suitable for the bio-medical field. Consequently, optimizing the performance in the non-federated conditions was not targeted, and improvements concerning state-of-the-art in this respect could not be demonstrated. However, making a federated architecture available enables the exploitation of multiple sources of unshared data that allows building on top of current cutting-edge single-institution solutions, overcoming the low data numerosity issue while improving the generalization ability of the overall system and naturally enabling multicentric studies. The proposed approach does not consider the problem of missing views, which also affects clinical data processing. However, we are confident that the openness and flexibility of the proposed approach will foster research in the field, marking a step in data sharing and distributed processing.

An additional limitation of the current study is the lack of an intermediate “validated federation” setting. This scenario would re-use the same 5-fold data split used to run the centralized experiments. Despite not being as realistic as the federated scenario presented here, it would add more comparable results between the centralized and federated settings and provide additional indicators to the current study.

Finally, a typical limitation of FL experiments is the need for huge amounts of memory. This problem can be emphasized when dealing with a simulated federation (i.e., all the clients span in the same device). Indeed, all the clients will own a copy of the neural network, and multiple copies of the same model can be problematic to handle for a single machine.

## Troubleshooting

The most common problem when running a real federation with OpenFL is the creation of the federation (protocol step 22).

### Problem 1

**Figure 7:**
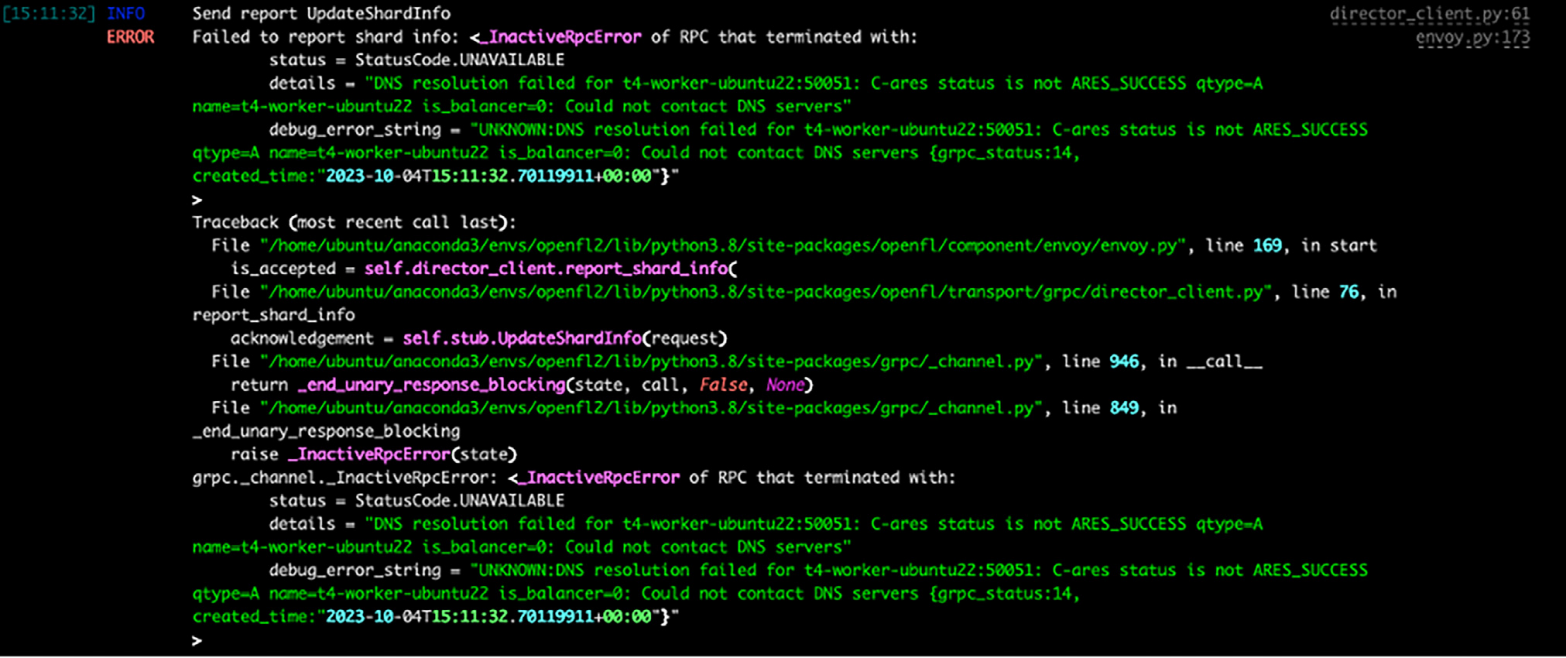
Wrong fully qualified domain name

The scripts for running the envoys do not contain the right FQDN of the director machine (Figure 7).

### Potential solution

• Double-check the FQDN of the director device.

>hostname -–fqdn

### Problem 2

**Figure 8:**
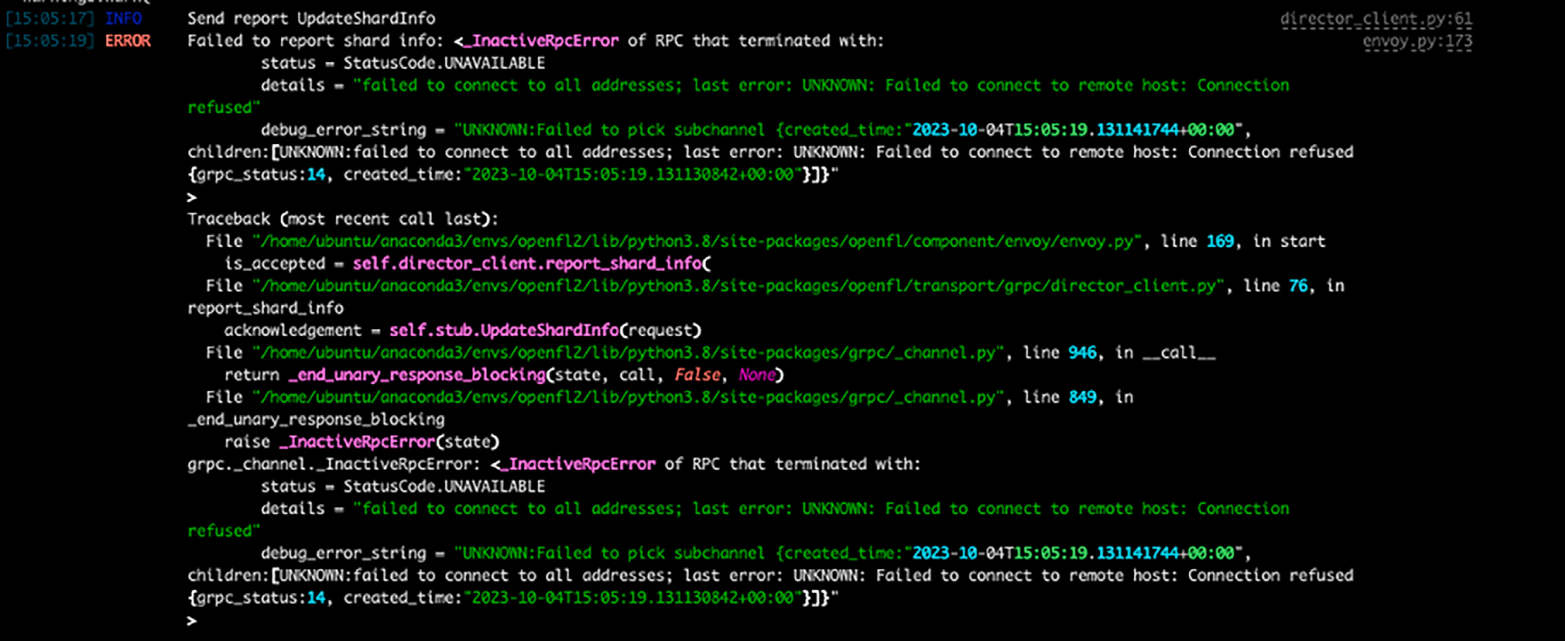
Envoy trying to connect to Director, before Director is alive

The envoy script has been executed before the director was alive (Figure 8).

### Potential solution

• Kill the alive envoys and restart the process paying attention to start the envoys only once the director is active.

**Figure 9:**
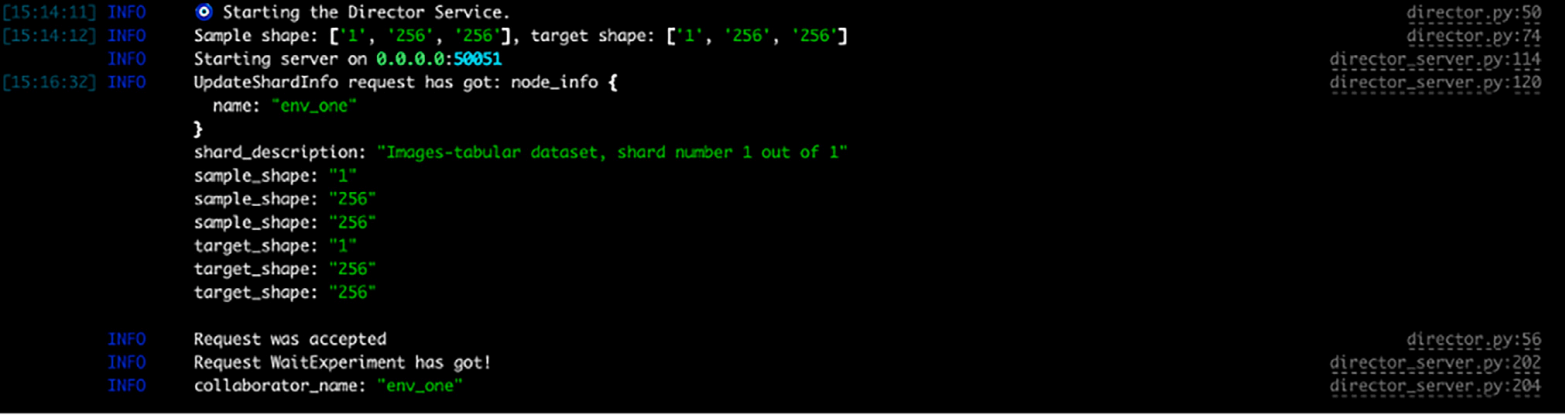
Director output when everything is set correctly

**Figure 10:**
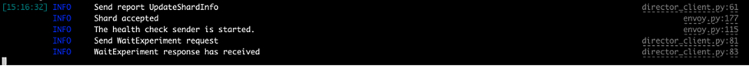
Envoy output when everything is set correctly

If the connection is initialized successfully, the director will print information about the envoy, while the envoy will idle until it receives an FL task to perform (Figures 9 and 10).

## Resource availability

### Lead contact

Request for information and resources used in this article should be addressed to Bruno Casella (bruno.casella@unito.it).

### Technical contact

Technical questions on executing this protocol should be directed to and will be answered by Bruno Casella (bruno.casella@unito.it).

### Materials availability

This study did not generate new unique reagents.

### Data and code availability

The code used for experimental evaluation is publicly available (see key resources table).

## Supplemental information

Data S1. train_testSET.zip contains a dummy dataset of the clinical parameters of the COVID dataset

## References

[1] Casella B., Riviera W., Aldinucci M., Menegaz G. (2023). MERGE: A Model for Multi-Input Biomedical Federated Learning. Cell Patterns.

[2] Soda P., D’Amico N.C., Tessadori J., Valbusa G., Guarrasi V., Bortolotto C., Akbar M.U., Sicilia R., Cordelli E., Fazzini D. (2021). AIforCOVID: Predicting the clinical outcomes in patients with COVID-19 applying AI to chest-X-rays. An Italian multicentre study. Med. Image Anal..

[3] Foley P., Sheller M.J., Edwards B., Pati S., Riviera W., Sharma M., Narayana Moorthy P., Wang S.H., Martin J., Mirhaji P. (2022). OpenFL: the open federated learning library. Phys. Med. Biol..

